# Single-cell and bulk RNA sequencing data jointly reveals *VDAC2’*s impacts on prognosis and immune landscape of NSCLC

**DOI:** 10.18632/aging.205517

**Published:** 2024-02-20

**Authors:** Ying Ma, Bateer Han, Qin Yu, Nashunbayaer Zha, Zhiyuan Deng, Junguo Liang, Rong Yu

**Affiliations:** 1 Department of Thoracic Surgery, Affiliated Hospital of Inner Mongolia Medical University, Hohhot 010059, Inner Mongolia Autonomous Region, China; 2 Department of Thoracic Surgery, Peking University Cancer Hospital (Inner Mongolia Campus) and Affiliated Cancer Hospital of Inner Mongolia Medical University, Hohhot 010020, Inner Mongolia Autonomous Region, China; 3 Department of Radiation Oncology, Peking University Cancer Hospital (Inner Mongolia Campus) and Affiliated Cancer Hospital of Inner Mongolia Medical University, Hohhot 010020, Inner Mongolia Autonomous Region, China

**Keywords:** non-small cell lung cancer, single-cell transcriptome, VDAC2, prognosis, immune landscape

## Abstract

Non-small cell lung cancer (NSCLC) is characterized by stronger metastatic ability and worse prognosis. In NSCLC, hypoxia is a major cause of invasion and metastasis through promoting angiogenesis. In present study, NSCLC cell clusters were extracted from single cell-sequencing dataset GSE131907, which were combined with hypoxia-related genes to group clusters. qRT-PCR and western blot were used to validate the expression of target gene. Nine NSCLC clusters were extracted, which were divided into two hypoxia-related subgroups, C1 and C2. Totally 101 differentially expressed prognostic genes were identified between subgroups. Of which, *VDAC2* showed excellent prognostic value for NSCLC and was selected for further analysis. *VDAC2* was upregulated in tumor samples in TCGA and was correlated with advanced stages. *In vitro* experiments validated this trend. Five crucial immune cells showed differential infiltration proportions between high and low *VDAC2* expression groups. *VDAC2* knockdown significantly inhibited the proliferation and invasion ability of NSCLC cells. Integrating single cell and bulk sequencing data as well as wet lab experiments, hypoxia-related *VDAC2* exhibited important prognostic value and showed the promise of becoming immune-therapy target in NSCLC.

## INTRODUCTION

Non-small cell lung cancer (NSCLC), as one of the main subtypes of lung cancer, accounts for 85% of all diagnosed lung cancer cases [1]. The primary subtypes of NSCLC comprise lung adenocarcinoma (LUAD) and lung squamous cell carcinoma (LUSC) [2]. Currently, in patients at stages I and II, the standard treatment of NSCLC is surgical resection followed by adjuvant systemic therapy [3]. In advanced NSCLC patients, the predominant treatment includes immune checkpoint inhibitor (ICI) monotherapy or its combination with chemotherapy [4]. Unfortunately, despite the great development of therapeutic strategies, the 5-year overall survival (OS) rate of NSCLC is still unsatisfied, approximately only 23% [5]. On the other hand, NSCLC is prone to metastasize at an early stage, especially lymph node metastasis, thus the prognosis of NSCLC is usually suboptimal [6]. Some markers, like PD-L1 expression and tumor mutation burden (TMB), have been applied in clinical lung cancer cases for predicting patients’ immunotherapy responses [7], whereas tumor heterogeneity is still a great challenge in clinical practice of cancers. Accordingly, great efforts should be devoted to exploring more details relating to the intratumoral heterogeneity of NSCLC, thereby indirectly conducive to improve the clinical outcome of patients.

Hypoxia is a common micro-environmental trait in most solid tumors, which is caused by an imbalance between tumor cell proliferation and blood oxygen supply [8]. Hypoxia in solid tumors has been linked to cancer patient resistance to chemotherapy and radiation, and it often promotes a more aggressive tumor phenotype, which contributes to poor outcomes of patients [9]. Hypoxia related conditions are increasingly regarded as the critical aspect affecting the malignant progression of lung cancer [10–12]. In NSCLC, long-term moderate hypoxia would elevate the expression of *FGFR1* and maintain the MAPK activation, thereby promoting epidermal growth factor receptor (EGFR) tyrosine kinase inhibitors (TKI) resistance [13]. Chronic intermittent hypoxia can promote lung cancer stem cells-like properties via activating mitochondrial reactive oxygen species (ROS) [14]. Hypoxia induction mediated by hypoxia-inducible factor 2α (HIF-2α) increases the epithelial-mesenchymal transition (EMT) events and *NEAT1* expressions in NSCLC under hypoxic condition via regulating miR-101-3p/SOX9/Wnt/β-catenin signal pathway, promoting the progression of NSCLC [15]. In addition, under hypoxic conditions, cell autophagy involves in improving NSCLC antiradiation capacity by regulating the amount of ROS, negatively affecting radiotherapy efficacy and leading to a poor prognosis of lung cancer patients [16]. In short, hypoxia can promote the NSCLC cell progression and chemoresistance. However, more detailed hypoxia related functions in heterogeneous NSCLC cells still lack clear clarification as far as we know.

Herein, in this study, the NSCLC related single-cell and bulk RNA sequencing data have been comprehensively analyzed in order to decipher more details involving the tumor microenvironment (TME) and intratumoral heterogeneity basing on hypoxia related genes. Our findings are promising to give more insights into understanding tumor as well as immune microenvironment of NSCLC patients.

## RESULTS

### Identification of heterogeneous NSCLC cell subpopulations

**Figure 1:**
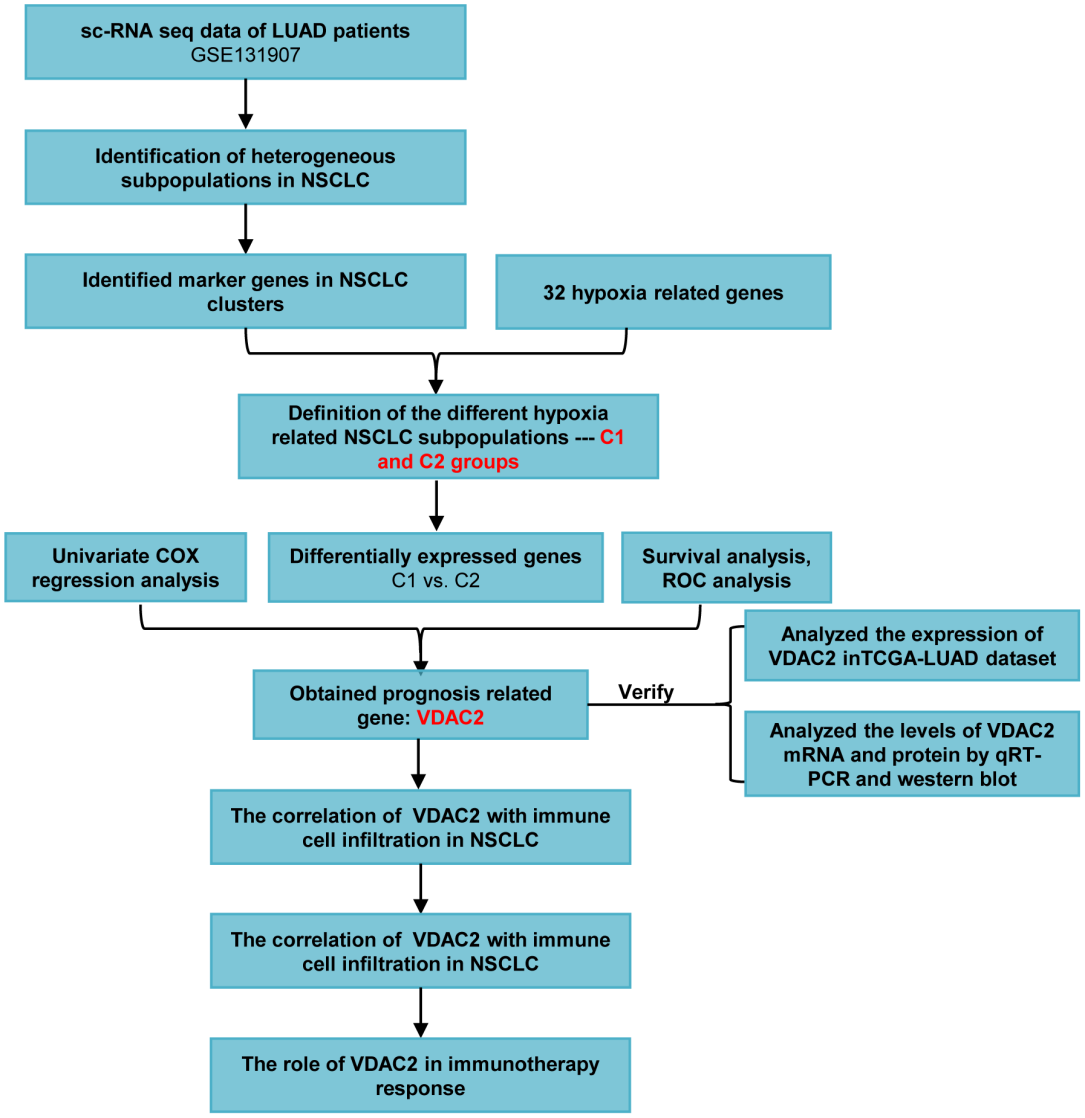
The flow chart of this study.

**Figure 2:**
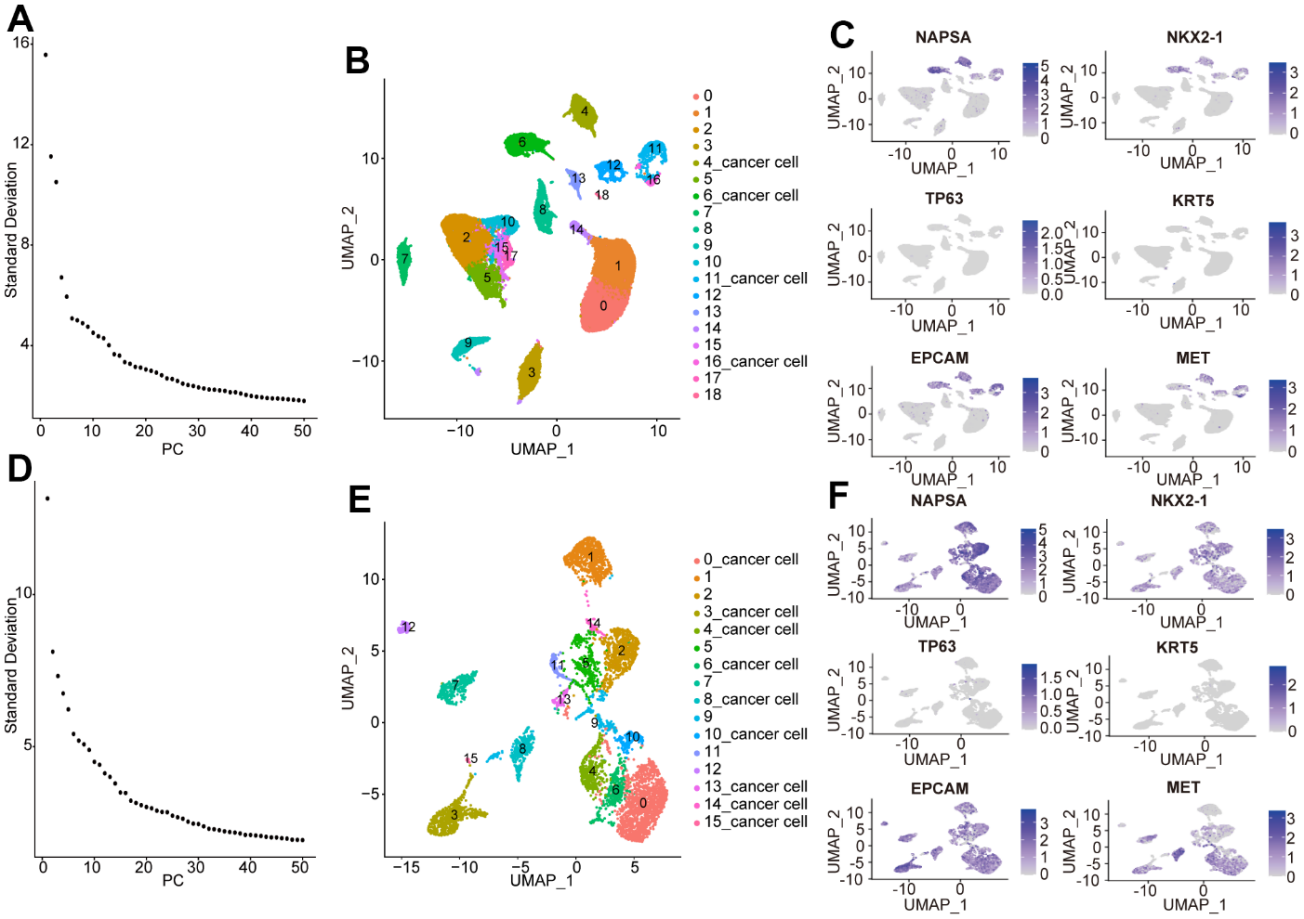
**Identification of heterogeneous NSCLC cell subpopulations.** (**A**) First principal component score, (**B**) The cells were annotated into 19 cell clusters by Umap in NSCLC. (**C**) Identified NSCLC clusters using marker genes of cancer. (**D**) Second principal component score. (**E**) The NSCLC cell populations were annotated into 16 cell clusters by Umap. (**F**) Identification of NSCLC cluster by cancer cell marker gene.

To better understand the intratumoral heterogeneity of NSCLC and hypoxia from distinct levels, our present work was conducted according to the flow chart in Figure 1. We firstly analyzed the sc-RNA sequencing data to identify the heterogeneous cell subsets. In GSE131907 dataset, we processed the samples with Seurat R package, and selected the top 15 principal components among all clusters (Figure 2A). The cells were annotated into 19 cell clusters by Umap (Uniform Manifold Approximation and Projection) method (Figure 2B). Subsequently, the LUAD marker genes *NAPSA*, *NKX2-1*, the LUSC marker genes *TP63*, *KRT5*, as well as *EPCAM* and *MET* were adopted to identify the cancer cell clusters. We found that cluster4, cluster6, cluster11, cluster12 and cluster16 belonged to cancer cell populations (Figure 2C). To screen the tumor cells more accurately, these 5 cancer cell clusters were analyzed following the above process again, and totally 16 cell clusters were obtained (Figure 2D, 2E). Finally, cluster0, cluster3, cluster4, cluster6, cluster8, cluster10, cluster13, cluster14, cluster15 were identified as the NSCLC cell populations by further cancer cell annotation (Figure 2F).

### Definition of different hypoxia related NSCLC subpopulations - C1 and C2 groups

Next, we analyzed the marker genes’ functional information of the above 9 cell subsets to better understand the potential functional ways. The marker genes of cluster0, cluster4 and cluster10 were significantly enriched in 9, 7 and 6 KEGG pathways, respectively, and the marker genes of other clusters were enriched in 20 KEGG pathways (Supplementary Figures 1, 2). In addition, the marker genes of these 9 clusters were significantly enriched in 20 GO terms, respectively (Supplementary Figures 3, 4). We noticed that most of the functional terms involved in multiple critical processes and aspects in tumorigenesis, such as MAPK signaling pathway and HIF-1 signaling pathway, which implied the distinct function of heterogeneous cell subsets in NSCLC.

**Figure 3:**
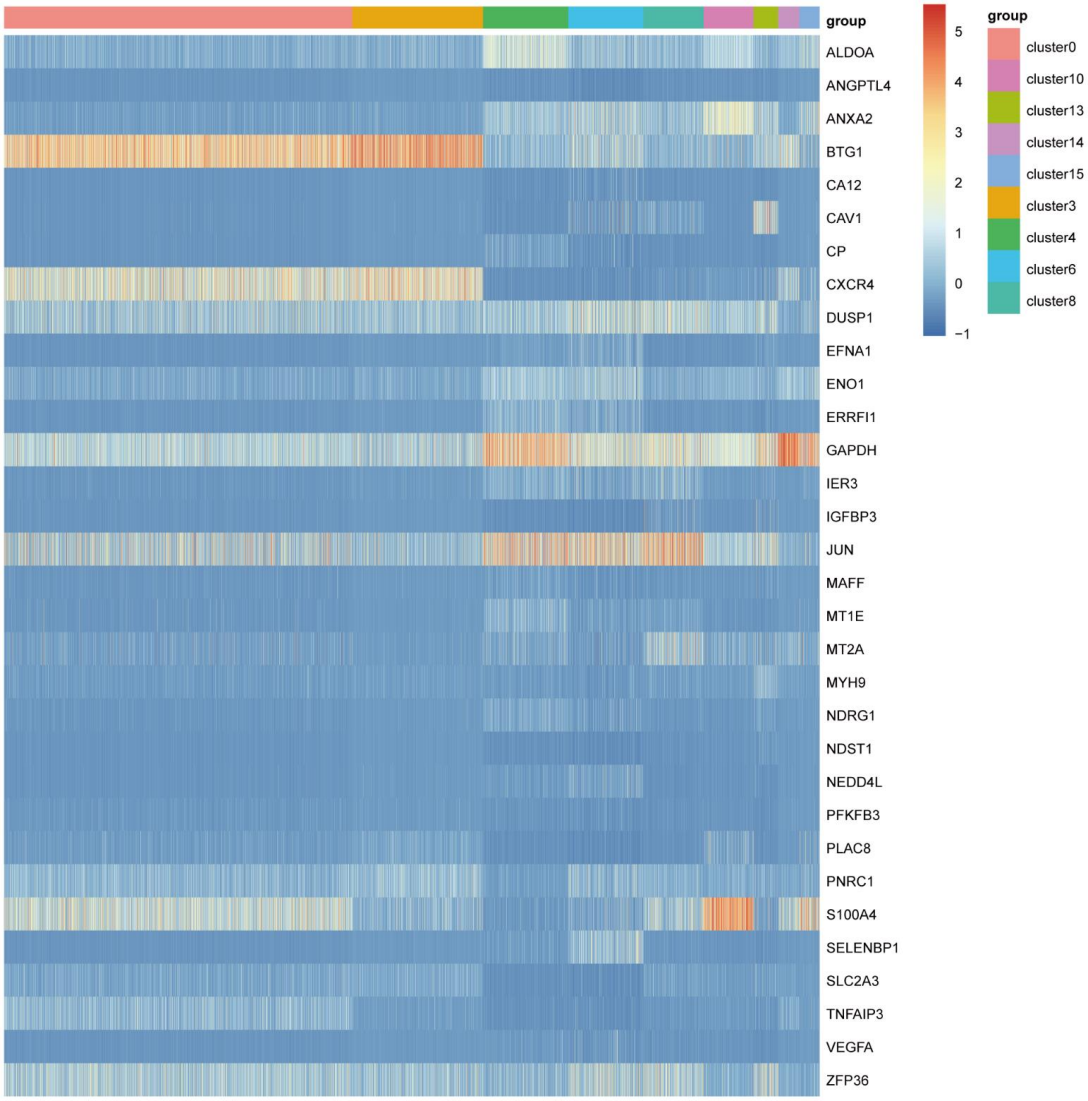
The heatmap of differential expression of hypoxia related marker genes in all cell clusters.

Hypoxic conditions in TME are considered as a driver of tumor malignancy in NSCLC [10]. To better decipher hypoxic impacts on tumor cells in TME, we performed the cross-over analysis between hypoxia related genes (HRGs) and marker genes of these cluster. The significantly differential HRG expression pattern was observed between cluster0, cluster3 and cluster4, cluster6, cluster8, cluster10, cluster13, cluster14 (Figure 3). Therefore, according to the HRG expression, the cluster0 and cluster3 were then defined as C1 group, and cluster4, cluster6, cluster8, cluster10, cluster13, cluster14 were classified as C2 group. The samples in C1 and C2 groups were considered distinct hypoxia related heterogeneous NSCLC subpopulations.

### Hypoxia heterogeneous NSCLC subpopulation related *VDAC2* showed important prognostic value in NSCLC

**Figure 4:**
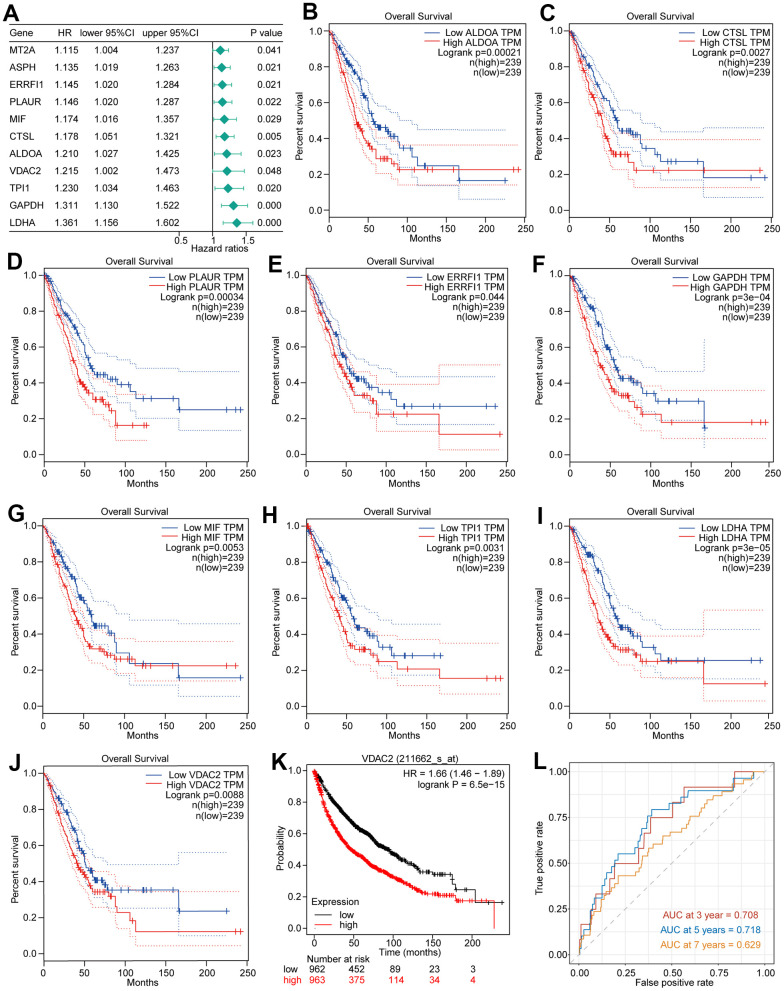
**Hypoxia heterogeneous NSCLC subpopulation related genes’ prognostic value in NSCLC.** (**A**) The 11 prognosis related genes with HR greater than 1. (**B**–**J**) The Kaplan Meier survival curves of 9 genes expression (high and low) in the TCGA-LUAD dataset. (**K**) The results of Kaplan Meier survival analysis of *VDAC2* in the 2437 NSCLC patients. (**L**) Time dependent ROC curves of *VDAC2* in NSCLC.

Among the marker genes in the hypoxia-related heterogeneous NSCLC subpopulations, C1 and C2, 101 genes were identified to be associated with prognosis of patients by univariate Cox regression analysis (Supplementary Table 1). Of these 101 genes, there were 11 genes with HR greater than 1, as risk factors for NSCLC (Figure 4A and Supplementary Table 1). Except for *ASPH*, *MT2A* (Supplementary Figure 5A, 5B), the other 9 genes’ high expressions were significantly correlated with a poor prognosis of NSCLC patients (Figure 4B–4J). Basing on the KM plotter (http://kmplot.com/) database, the *VDAC2* high expression was significantly associated with inferior prognosis of 2437 NSCLC patients among these 9 genes (Figure 4K). *VDAC2* expression exhibited significantly positive correlation with HIF-1α (Supplementary Figure 5C). Moreover, the time dependent receiver operating characteristic (ROC) indicated that area under the curve (AUC) values of *VDAC2* in 3 years and 5 years were over 0.70, and the AUC value of *VDAC2* in 7 years was 0.629 (Figure 4L), indicating *VDAC2* had better diagnostic value for NSCLC. Therefore, *VDAC2* was selected for our subsequent analysis.

### *VDAC2* was highly expressed in NSCLC

**Figure 5:**
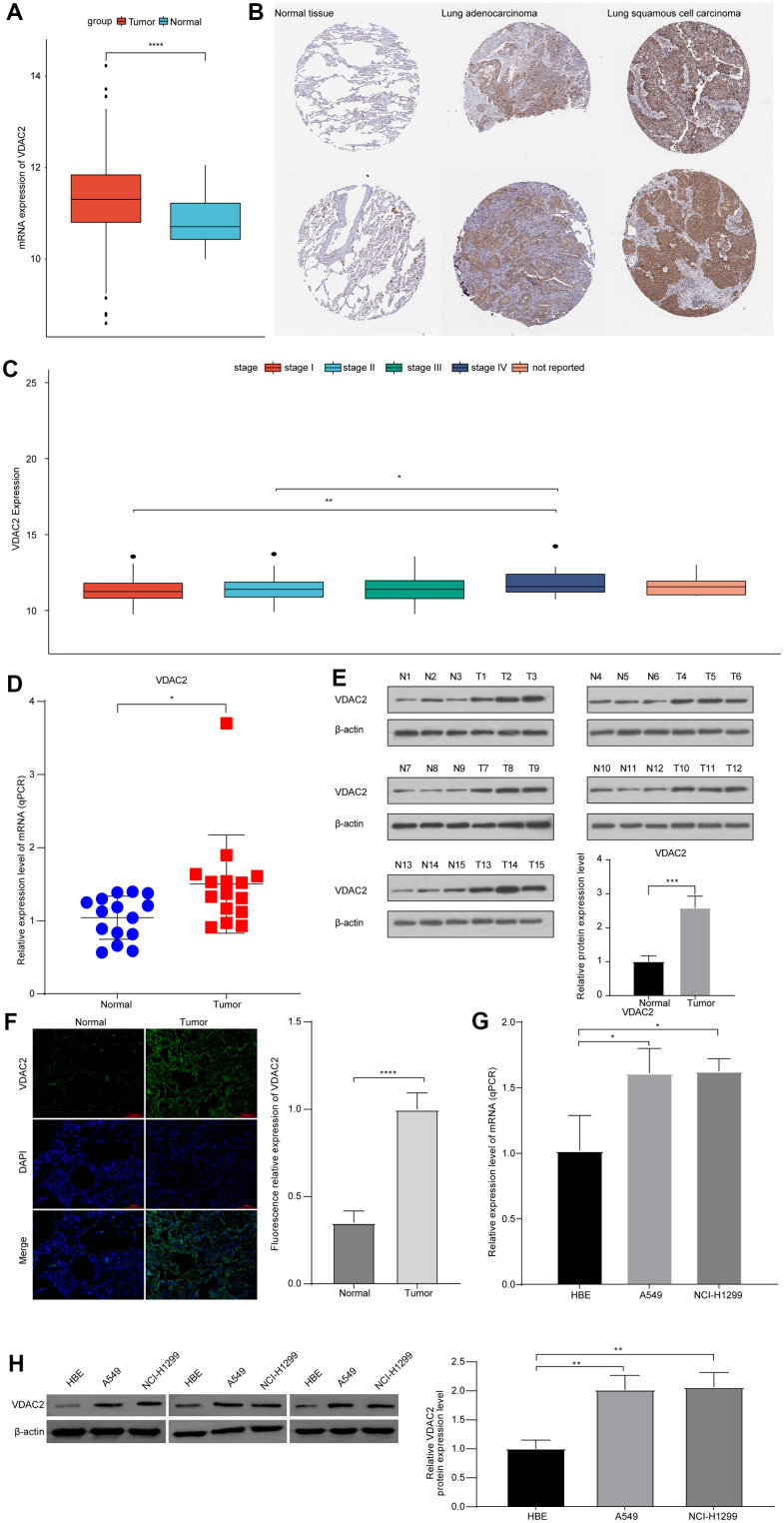
**Hypoxia heterogeneous NSCLC subpopulation related *VDAC2* showed important prognostic value and tumor promoting role in NSCLC.** (**A**) The expression of *VDAC2* in normal and cancer samples basing on TCGA-LUAD cohort. (**B**) The expression of *VDAC2* in NSCLC tissues and normal tissues basing on Human protein atlas database. (**C**) The expression of *VDAC2* in different stages of NSCLC patients. (**D**, **E**) The levels of *VDAC2* mRNA and protein in clinical NSCLC tissues. (**F**) The expression of *VDAC2* in lung cancer and adjacent noncancerous tissues was detected by immunofluorescence. (**G**, **H**) The levels of *VDAC2* mRNA and protein expression in A549 and NCI-H1299 cells. * *p*<0.05, ** *p*<0.01, **** *p*<0.0001.

In the TCGA-LUAD dataset, we found that the *VDAC2* was significantly highly expressed in cancer samples comparing to adjacent samples (Figure 5A, cancer vs. normal). A similar high expression of VDAC2 protein was also observed in tumor tissue samples of NSCLC from Human Protein Atlas (HPA, https://www.proteinatlas.org/) database (Figure 5B). Furthermore, the expression of *VDAC2* was significantly increased in stage IV patients comparing to stage I and stage II patients (Figure 5C).

To validate the *VDAC2* expression in NSCLC using wet lab tools, we analyzed the expression of *VDAC2* in NSCLC clinical samples and cell lines. The levels of *VDAC2* mRNA and protein were increased in NSCLC tissue comparing to normal tissue (Figure 5D, 5E). Immunofluorescence results showed that VDAC2 protein expression was significantly increased in lung cancer tissues compared to adjacent noncancerous tissues (Figure 5F). Moreover, VDAC2 mRNA and protein were also highly expressed in A549 and NCI-H1299 cells compared to HBE cells (Figure 5G, 5H). Collectively, the aberrant expression of *VDAC2* might show tumor promoting role in NSCLC.

### The correlation between *VDAC2* and immune landscape in NSCLC

**Figure 6:**
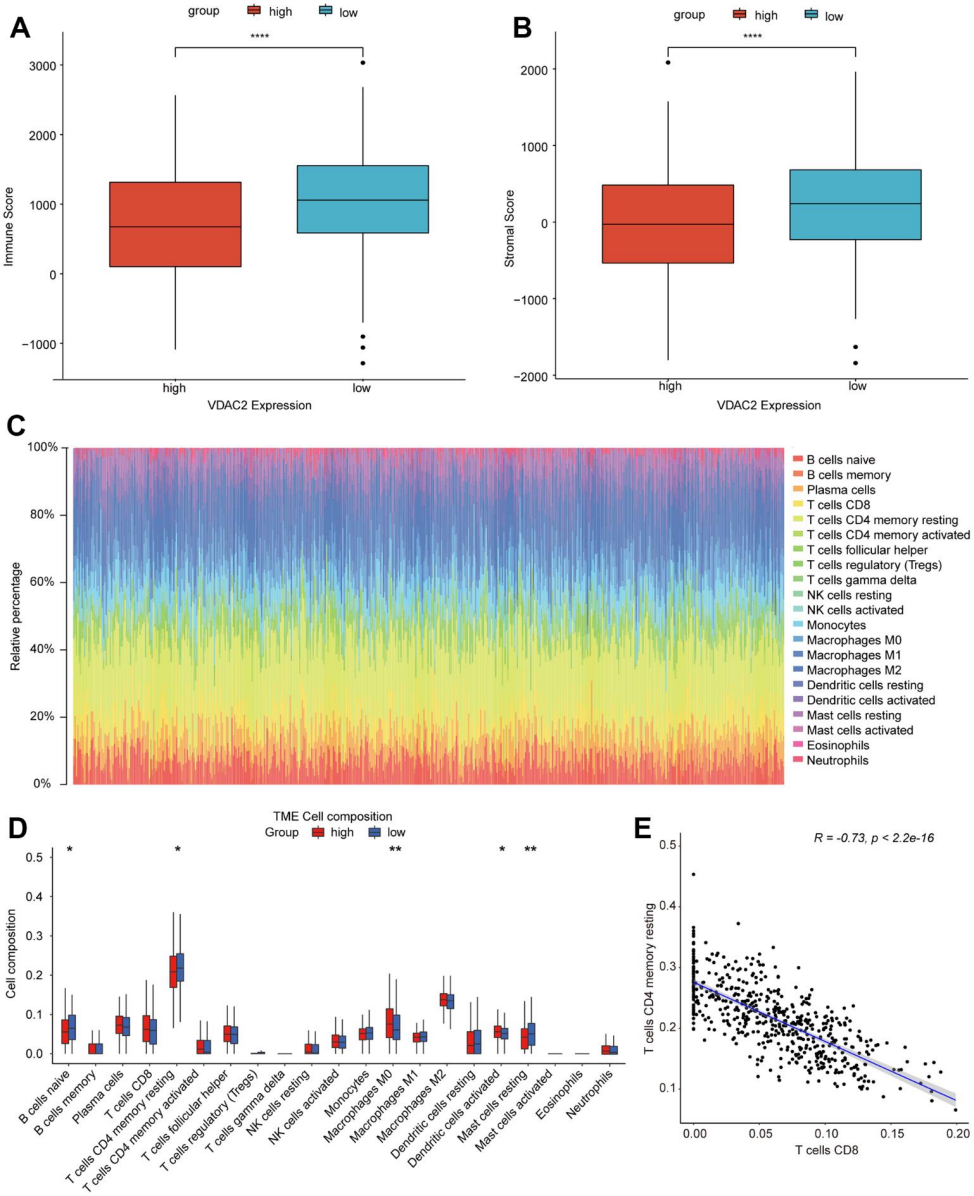
**The correlation between *VDAC2* and tumor microenvironment in NSCLC.** (**A**, **B**) The stromal score (**A**) and immune score (**B**) between high and low *VDAC2* expression groups. (**C**, **D**) The correlation of *VDAC2* with immune cells infiltration. (**E**) The correlation between T cells CD8 and T cells CD4 memory resting in NSCLC. * *p* <0.05, ** *p*<0.01; *** *p*<0.001, **** *p*<0.0001.

The samples in TCGA-LUAD dataset were spilt into high and low *VDAC2* expression groups according to the median value of *VDAC2* expression. Firstly, the immune score and stromal score were decreased in high *VDAC2* expression group (Figure 6A, 6B). In addition, comparing to low *VDAC2* expression group, the relative infiltrating proportions of B cells naïve, T cells CD4 memory resting, Mast cells resting were reduced in high *VDAC2* expression group, and the Macrophages M0, Dendritic cells activated were increased (Figure 6C, 6D). Among all differentially infiltrated immune cells, the correlation analysis indicated that the T cells CD8 had strong negative correlation with T cells CD4 memory resting in NSCLC (Figure 6E).

### NSCLC patients’ clinical responses with distinct *VDAC2* expression

**Figure 7:**
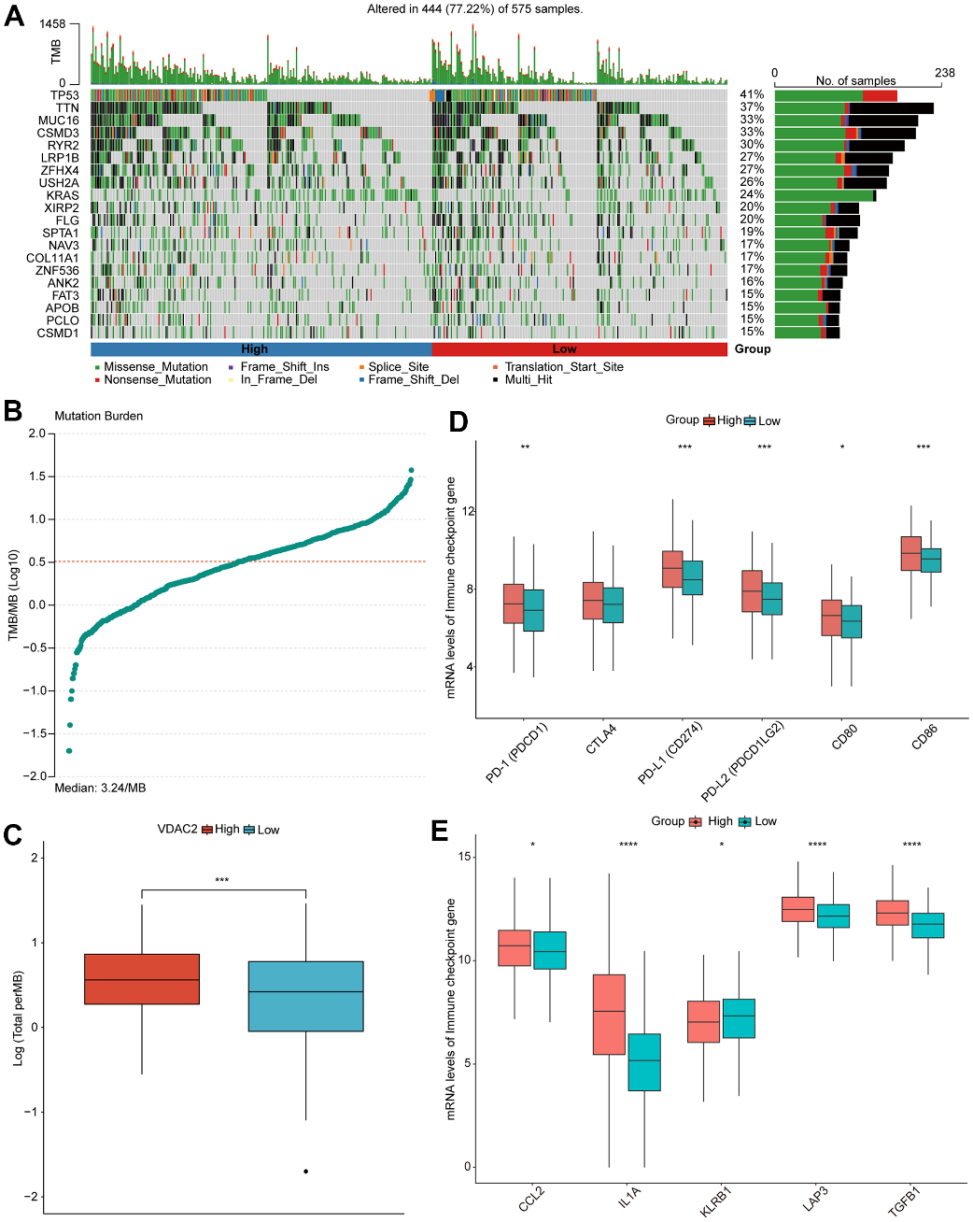
**NSCLC patients with distinct *VDAC2* expression exhibited different clinical responses.** (**A**) The waterfall plot of somatic mutation. (**B**, **C**) The level of TMB in high and low *VDAC2* expression groups. (**D**, **E**) The expression of immune checkpoints in high and low *VDAC2* expression groups. * *p* <0.05, ** *p*<0.01; *** *p*<0.001.

Subsequently, we analyzed the somatic mutation status in high and low *VDAC2* expression groups, and calculated the TMB. The *TP53* and *TTN* were more frequently mutated in high *VDAC2* expression group than in low expression group (Figure 7A). In addition, the TMB was significantly increased in high *VDAC2* expression group (Figure 7B, 7C). Critical immune checkpoints PD-1 *(PDCD1)*, PD-L1 *(CD274)*, PD-L2 *(PDCD1LG2)*, *CD80*, *CD86* were significantly upregulated in high *VDAC2* expression group (Figure 7D). The immune checkpoints *CCL2*, *IL1A*, *LAP3* and *TGFB1* expressions were significantly increased in high *VDAC2* group, whereas *KLRB1* expression was decreased (Figure 7E, high vs. low).

### Suppression of *VDAC2* expression might inhibit proliferation and invasion of NSCLC cells *in vitro*

**Figure 8:**
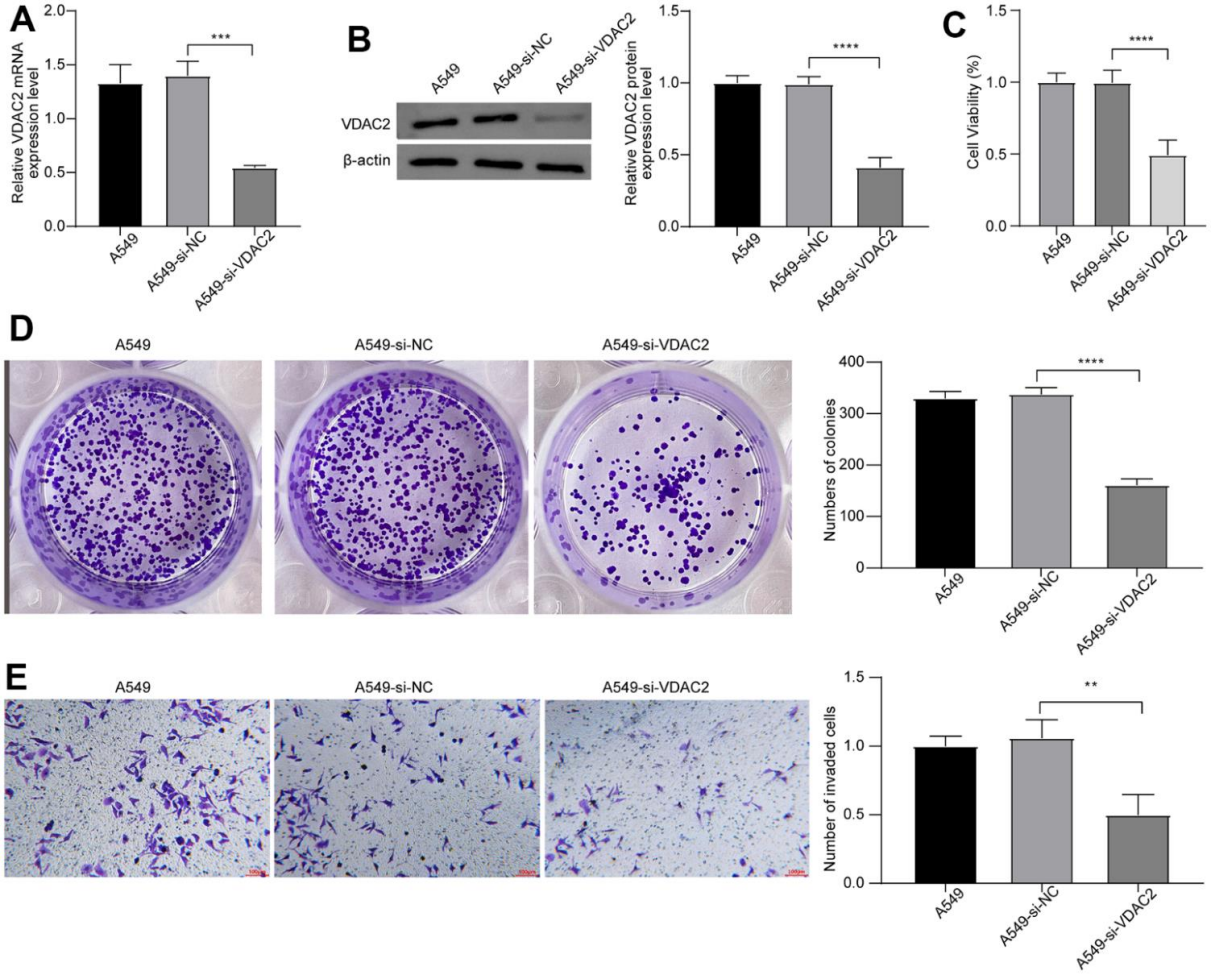
**Suppression of *VDAC2* expression might inhibit proliferation and invasion of NSCLC *in vitro*.** The levels of *VDAC2* mRNA expression (**A**) and protein expression (**B**) in A549-si-*VDAC2* group detected via qPT-RCR and western blot. (**C**) Cell viability was measured by CCK8 assay. (**D**) Cell proliferation was detected by clonogenic assay. (**E**) Invasion ability of cells was detected via transwell assay. ** *p*<0.01; *** *p*<0.001, **** *p*<0.0001.

To further decipher the effects of *VDAC2* on progression of NSCLC, we constructed the *VDAC2* knockdown (si-*VDAC2*) A549 cells, and investigated the effects of *VDAC2* underexpression on proliferation and invasion of NSCLC cells. The levels of *VDAC2* mRNA and protein expressions were significantly decreased in A549-si-VDAC2 group compared to A549-si-NC group (Figure 8A, 8B). The CCK-8 assay showed that knockdown of *VDAC2* significantly inhibited the viability of A549 cells compared to empty vector group (si-NC) (Figure 8C). The results from the clonogenic assay were consistent with the CKK-8 assay data, suggesting that suppression of *VDAC2* expression inhibited proliferation of A549 cells (Figure 8D). In addition, the result of the transwell assay showed that knockdown of *VDAC2* could attenuate the invasion ability of A549 cells (Figure 8E). These results indicated that *VDAC2* knockdown could inhibit proliferation and invasion ability of NSCLC cells *in vitro*.

## DISCUSSION

In this study, we identified nine clusters as NSCLC cell populations by cancer cell identification and signature analysis. Moreover, according to the hypoxia related genes’ expression, these clusters were divided into two subgroups (C1 and C2). Totally 101 marker genes of C1 and C2 groups were significantly associated with prognosis of NSCLC patients. Of these 101 genes, high *VDAC2* expression was significantly associated with inferior prognosis of 2437 NSCLC patients, meanwhile *VDAC2* exhibited tumor promoting potential in NSCLC. *VDAC2* is one of the isoforms of Voltage Dependent Anion Channel (VDAC), and it has been proved to be ubiquitously expressing in mammalian [17]. VDAC exists in three isoforms, VDAC1, VDAC2 and VDAC3 [18]. It has been demonstrated that VDAC plays important role in regulating mitochondrial metabolism and energy functions, and it is involved in apoptotic machinery [16, 19]. Jóźwiak at al. have revealed that the high level of VDAC2 protein indicated a poor prognosis of endometrial cancer patients [20], which was consistent with our findings that NSCLC patients with overexpression of *VDAC2* had worse prognosis. We also found that higher *VDAC2* expression was correlated with advanced NSCLC stages. However, Zhou et al. have demonstrated that the patients with *VDAC2* overexpression had better prognosis in glioma, and the *VDAC2* expression was inversely correlated with grades [21]. The above evidences showed that *VDAC2* probably exhibited distinct functions in different cancer types. In addition, *VDAC2* plays a critical role in regulating BAX mediated apoptosis [22]. Wang et al. have reported that the knockdown of *VDAC2* increased the mitochondrial membrane potential and total ROS levels in pancreatic ductal adenocarcinoma cells, and *VDAC2* knockdown potentiated the apoptosis induced by chemotherapy [23]. In melanoma cells, the *FOXM1* could inhibit ferroptosis via regulating the expression of *Nedd4* and the degradation of *VDAC2/3* [24]. In this study, we found that *VDAC2* knockdown significantly inhibited the proliferation and invasion ability of NSCLC cells *in vitro*. However, more details involving how *VDAC2* regulated the malignant cell events in NSCLC deserved further illustration in our future work.

Additionally, the relative proportions of B cells naïve, T cells CD4 memory resting, Mast cells resting were reduced in high *VDAC2* expression group, and the Macrophages M0, Dendritic cells activated were increased. These results indicated that the B cells naïve, T cells CD4 memory resting and Mast cells resting were associated with better prognosis of NSCLC patients, and Macrophages M0, Dendritic cells activated were correlated with poor prognosis. B cells and T cells were integral part of tumor infiltrating lymphocytes. In addition, the T cells predominated in lung cancer immune landscape, and CD4+ T cells and CD8+ T cells were the common prevalent T cell subtypes [25]. The high levels of CD3+ and CD8+T cells have been implied to correlate with longer survival rate of NSCLC patients [26], and the NSCLC patients with higher CD8(+) counts had longer OS rate [27]. These evidences suggested that the higher CD8(+) infiltration had better prognostic value in lung cancer, while it was not observed in our present analysis. In addition, Gentles et al. have found that the inactivated mast cells and inactivated CD4 memory T cells were strongly correlated with better prognosis of LUAD patients [28], which indirectly supported our results. In addition, dendritic cells belong to a heterogeneous population of leukocytes that present tumor antigens to antigen-specific helper and cytotoxic T cells [29]. Got et al. have revealed that the mature dendritic cells high density exhibited a strong T cell infiltration in NSCLC patients, and the density of mature dendritic cells was associated with the expression of T-cell activation related genes [30]. They also found that the elevated density of dendritic cells was associated with longer survival rate of NSCLC patients. Accordingly, we hypothesized that the high *VDAC2* expression might be involved in the immune cell infiltration, whereby contributing to the worse prognosis of NSCLC patients.

We also found that the TMB was significantly increased in high *VDAC2* expression group. TMB is a measure of the total number of mutations per coding area of tumor genome, which has been reported to be correlated with the objective response rate to immunotherapy (PD-1 inhibitors) [31]. Previous studies have documented that the high TMB and PD-L1 expression were correlated with better benefit from immune checkpoint blockade treatment in NSCLC patients [32, 33]. Of note, we found that the *PD-1*, *PD-L1*, *PD-L2*, *CD80*, *CD86* were upregulated in high *VDAC2* expression group. Above data suggested that the *VDAC2* expression was indirectly associated with TMB status and immune checkpoint expressions in NSCLC patients, which provided important reference for immunotherapy of NSCLC.

Although our study identified different hypoxia related NSCLC subpopulations - C1 and C2 groups, and obtained a prognosis-related gene *VDAC2* in NSCLC, certain limitations should be noted. On the one hand, the correlation of *VDAC2* expression and cell hypoxia in NSCLC needs further investigations via hypoxia related cell experiments. On the other hand, the NSCLC patients’ clinical responses with distinct *VDAC2* expression should be validated in clinical trial cohort, which would further strengthen the clinical predictive value of *VDAC2* in NSCLC.

In summary, basing on hypoxia related heterogeneous NSCLC subpopulations, the prognostic value of *VDAC2* in NSCLC was demonstrated in our work for the first time, integrating single cell sequencing data, bulk sequencing data, and wet lab experiments. High *VDAC2* expression was an unfavorable prognostic indicator for NSCLC patients, which was profoundly correlated with TMB and immune checkpoints in NSCLC patients. Our findings are expected to provide more reference for the clinical management of patients.

## MATERIALS AND METHODS

### Data collection

The single-cell RNA sequencing (sc-RNA seq) GSE131907 dataset was collected from Gene Expression Omnibus (GEO, https://www.ncbi.nlm.nih.gov/geo/) database. This dataset included 58 clinical samples (comprising 15 lung tumor samples, 5 pleural fluid samples, normal samples and other tissue samples) of 44 LUAD patients. The mRNA expression profiles and somatic mutation data of 513 samples were downloaded from The Cancer Genome Atlas (TCGA, https://tcga-data.nci.nih.gov/tcga/) database. Of these 513 samples, totally 500 samples had complete survival information. In addition, the immunohistochemical data of target gene in NSCLC were obtained from the Human Protein Atlas (HPA, https://www.proteinatlas.org/) database.

### Sample source

Totally 15 NSCLC tissues and 15 normal tissues were obtained from the Affiliated People’s Hospital of Inner Mongolia Medical University. All experiments were approved by the ethics committee of the Affiliated People’s Hospital of Inner Mongolia Medical University (ethic code: No. WZ202307), conformed to the declaration of Helsinki, and informed consent was obtained from all subjects. The information of patients was shown in Supplementary Table 2.

### Identification of heterogeneous subpopulations in NSCLC

The R package “Seurat” was used for filtering out low-quality cells, data normalization, data dimension reduction and other processing of sc-RNA sequencing data in GSE131907 dataset. Then, the cancer cell populations were extracted and further processed again, and all tumor cells were divided into distinct cell clusters.

### Identification of hypoxia related NSCLC subpopulation

All of the obtained tumor cell subsets underwent the marker gene annotation, with *p* < 0.05 as a screening criterion. The marker genes were subjected to Gene ontology (GO) and Kyoto Encyclopedia of Genes and Genomes (KEGG) enrichment analysis by R (version 4.2.0, the same below) package “clusterProfiler”. The enriched pathways were selected using *p* < 0.05.

Moreover, we had also downloaded 32 HRG from gene set enrichment analysis (GSEA) database (https://www.gsea-msigdb.org/gsea/index.jsp). A cross analysis was then conducted between the marker genes and the HRGs to obtain the candidate genes.

### Screening of prognostic candidate genes

Based on the expressions of marker genes and HRGs, the cell clusters were split into C1 and C2 groups. The differentially expressed genes (DEGs) between these two groups were screened using R language package limma, based on |Log_2_FC|>0.5 and FDR<0.05. The obtained DEGs were subjected to univariate Cox regression and survival analysis, to further optimize the candidate genes. The survival analysis was conducted using Kaplan-Meier method. Time dependent receiver operating characteristic (ROC) analysis was used to predict the prognostic value of hub gene, in timeROC package of R language.

### Gene expression analysis

Gene expression matrix of LUAD samples (TCGA-LUAD.htseq_counts.tsv.gz) were downloaded from the GDC TCGA-LUAD cohort. This matrix included the expression values of VDAC2. Subsequently, the Wilcoxon rank sum test was used to compare the expression of *VDAC2* in different groups. *P* < 0.05 is considered statistically significant.

### Immune cell infiltration analysis

Estimation of STromal and immune cells in MAlignant Tumour tissues using Expression data (ESTIMATE) (https://R-Forge.R-project.org/projects/estimate/) function package inferred the proportion or abundance of tumor cells, infiltrating immune cells and stromal cells using transcriptional profiles of cancer samples to calculate the stromal score and immune score of the tumor tissues [34]. The TMB was calculated using R package “maftools”. The infiltration proportions of 22 kinds of immune cells between two groups were analyzed using CIBERSORT [35]. CIBERSORT investigators first designed and validated a leukocyte gene signature set LM22 comprising 574 genes that could distinguish 22 hematopoietic phenotypes, including 7 types of T cell, Naïve and memory B cell, plasma cell, NK cell, and myeloid subsets. The relative proportions corresponding to the 22 immune cells were calculated by deconvolution algorithm based on LM22 pair tissue RNA sequencing data.

### Cell culture

The cell lines used in this study included human bronchial epithelial like HBE cells, human NSCLC cells A549 and NCI-H1299. All cells were purchased from Hunan Fenghui Biological Co., Ltd. (Hunan, China). The HBE, A549 and NCI-H1299 cells were cultured in DMEM medium (Gibco, C11995500BT), Ham’s F-12K medium (Gibco, 21127030) and RPMI Medium 1640 medium (GIBCO, C11875500BT), respectively. All mediums were supplemented with 10% fetal bovine serum (GIBCO, 10270-106) and penicillin streptomycin mix (Solarbio, p1400), and placed in 37° C incubator with 5% CO_2_.

### qRT-PCR assay

The total RNA was extracted with Redzol Reagent (SBS, FTR-50). Reverse transcription was performed with a reverse transcription Kit (GeneCopeia, QP056). The PCR amplification was performed with 2 × SYBR Green qPCR Master Mix (None Rox) (Servicebio, Mpc2203026) in an iQ5 Real-Time PCR amplicon (Applied Biosystems). The primer sequences were displayed in Table 1. The program was as follows: 40 cycles at 95° C for 30 s, 95° C for 15 s, 60° C for 30 s. The internal reference was *β-actin*. mRNA expression levels were determined with 2^-ΔΔCT^ formula.

**Table 1:** Primer sequences for qPCR.

**Target gene**	**Primer sequence**
VDAC2-F	CGGAATTCATGGCGACCCACGGACAG
VDAC2-R	CTAGCTAGCAGCCTCCAACTCCAGGGCG
β-actin-F	CCACGAAACTACCTTCAACTCC
β-actin-R	GTGATCTCCTTCTGCATCCTGT

### Western blot assay

The protein was extracted using RIPA lysis solution (Beijing Solarbio Technology Co., Cat # R002) and PMSF solution. The western blot was consistent with previous methods [36]. The first antibodies included VDAC2 (9412, 1: 1000, Cell signaling technology), β-actin (ab8226, 1: 1000, Abcam). The second antibody was HRP-labeled Goat anti-rabbit IgG (ab7090, 1: 8000, Abcam). The gray values of the bands were analyzed using ImageJ software.

### Immunofluorescence assay

The lung cancer tissues and adjacent normal tissues were collected to make paraffin sections. The paraffin sections were put into sodium citrate antigen retrieval solution and placed in a microwave oven for 2 h at a temperature of 67° C. After recovery to room temperature, the sections were washed three times with PBS (pH7.4) for 5 min each time. Then, 50  μL 3% H_2_O_2_ was added and incubated at the room temperature for 30 minutes, and rinsed three times with PBS for 5 min each time. The sections were subsequently incubated with 0.5%Trition X-100 for 8 min at the room temperature, and rinsed three times with PBS for 5 min each time. Moreover, the sections were blocked with 5% goat serum for 60 min at room temperature, and then incubated with VDAC2 antibody (ab155803, 1:500, Abcam) overnight at 4° C, and rinsed three times with PBS for 5 min each time. Then, HRP-labeled Goat anti-rabbit IgG (ab7090, 1:8000, Abcam) was used to incubated sections for 2 h, and rinsed three times with PBS. Finally, the sections were blocked with an anti-fluorescence quencher, and observed under a fluorescence microscope.

### Transfection assay

Specific siRNAs targeting *VDAC2* (si-*VDAC2*) and control siRNA (si-NC) were purchased from Guangzhou RiboBio Co., Ltd. (Guangzhou, China). The A549 cells were inoculated in six-well plates and cultured at 37° C for 24 h with 5% CO2. Subsequently, the cells were transfected with si-*VDAC2* and si-NC using Lipofectamine® 3000 (2343152, Invitrogen) at 37° C for 48 h according to the manufacturer’s protocol. After 48 h transfection, qRT-PCR and western blotting were used to detect transfection efficiency.

### Cell proliferation

The cell counting kit-8 (CCK-8) assay kit (AR1160-100, Boster Biological Technology., LTD, Wuhan, China) was used to analyze the cell proliferation capacity. Log phase cells were collected and the concentration of cell suspension was adjusted, and 100 μL cell suspensions were added into 96-well plates (2×10^3^ cells/well). Then the plates were pre-incubated in an incubator at 37° C with 5% CO_2_. After 24 h, the 10 μL CCK-8 solution was added to each well and incubated for 1 h. The absorbance was measured with Microplate Reader (DNM-9602, Beijing Perlong Technology Co., Ltd, Beijing, China) at 450 nm.

### Transwell migration assay

The transwell chamber was placed into a 24-well plate and coated with 100 μL Matrigel (300ug/mL) onto the compartment membrane, and the gel was placed in an incubator at 37° C for 3 h. The 200 μL cell suspensions (1~5×10^5^ cells/mL) were added to the upper chamber and 500 mL of complete medium was added to the lower chamber at 37° C and 5% CO_2_ in the incubator for 24 h. Next, transwell chamber was washed twice with PBS and fixed with 4% paraformaldehyde for 30 min and stained with dye crystal violet solution for 30 min. The cells were washed with PBS and photographed using an inverted microscope (Olympus Corporation).

### Clonogenic assay

The cells were seeded into 12 well microtiter plates at a density of 500 cells/well to a total volume of 200 μL per well, and incubated at 37° C in for 1 week. Then, the cells were washed with PBS (pH7.4), and were fixed with 1 mL 4% paraformaldehyde for 30 min, and washed with PBS. Next, the cells were stained with 1 mL of 0.1% crystal violet for 30 min, and rinsed with PBS. The plate was allowed to dry naturally and photographed. The number of colonies was obtained by analyzing the digital photographs of the plates, using the program ImageJ.

### Statistical analysis

All bioinformatics data were analyzed using R software (Version 4.2.1). All experimental data were presented as mean ± standard deviation (SD). Data were analyzed using GraphPad Prism 6. Comparisons between multiple groups were made with one-way ANOVA, comparisons between two groups were performed by Independent-samples t test. The *p* < 0.05 was considered statistically significant.

### Availability of data and materials

The datasets generated and analyzed during the current study are available from the Gene Expression Omnibus (GEO) datasets (http://www.ncbi.nlm.nih.gov/geo) and The Cancer Genome Atlas (TCGA, https://tcga-data.nci.nih.gov/tcga/) database.

## Supplementary Material

Supplementary Figures

Supplementary Tables
