# Supplementary material for: Single-cell and bulk RNA sequencing data jointly reveals VDAC2’s impacts on prognosis and immune landscape of NSCLC
Source: Aging (Albany NY). 2024 Feb 20;16(4):3160–84. doi: 10.18632/aging.205517 (PMC10929798; doi:10.18632/aging.205517)
Supplement: Supplementary Figures [file aging-16-205517-s001.pdf]

SUPPLEMENTARY FIGURES

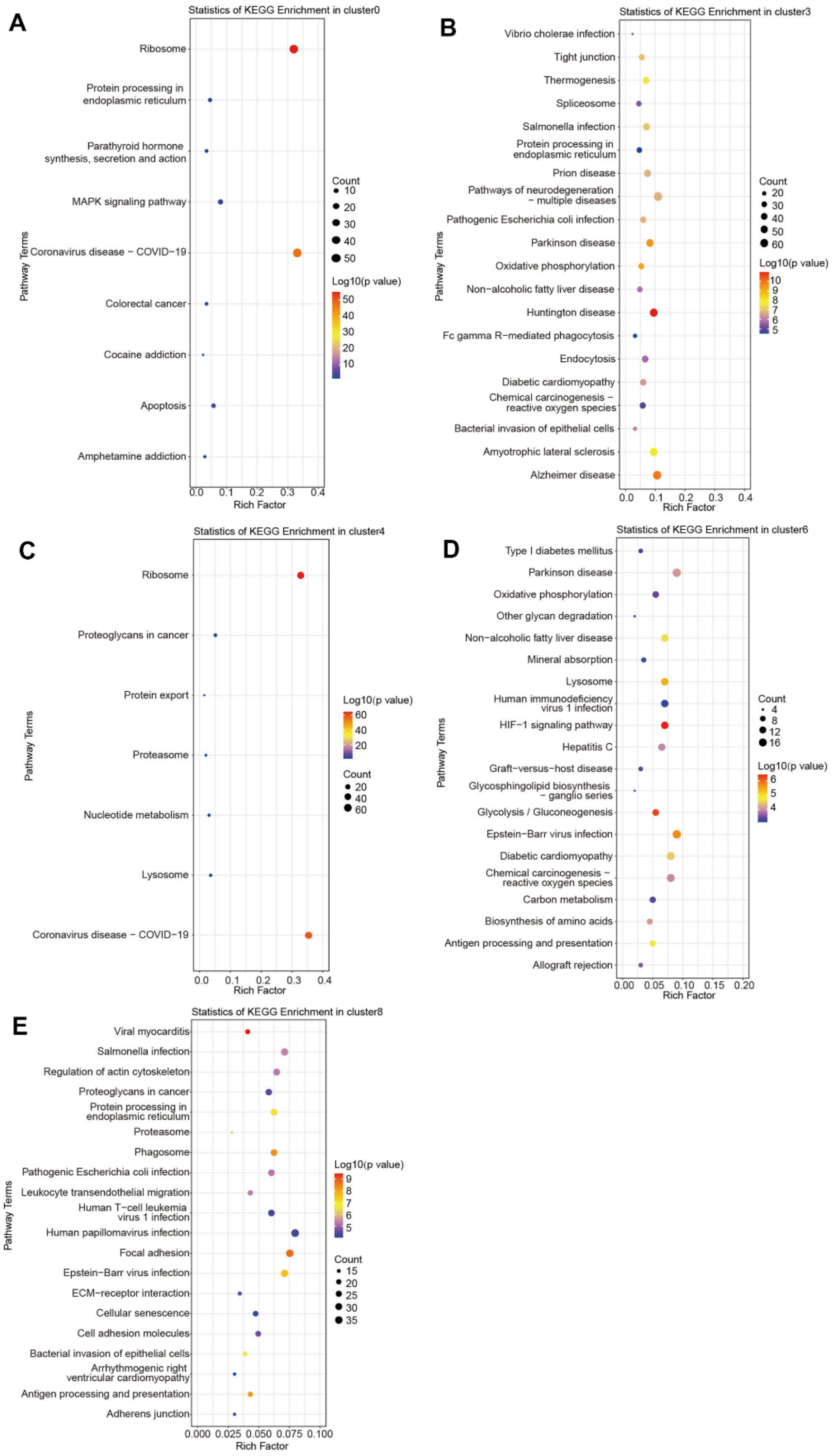

Supplementary Figure 1. The results of KEGG enriched analysis in cluster0 (**A**), cluster3 (**B**), cluster4 (**C**), cluster6 (**D**) and cluster8 (**E**).

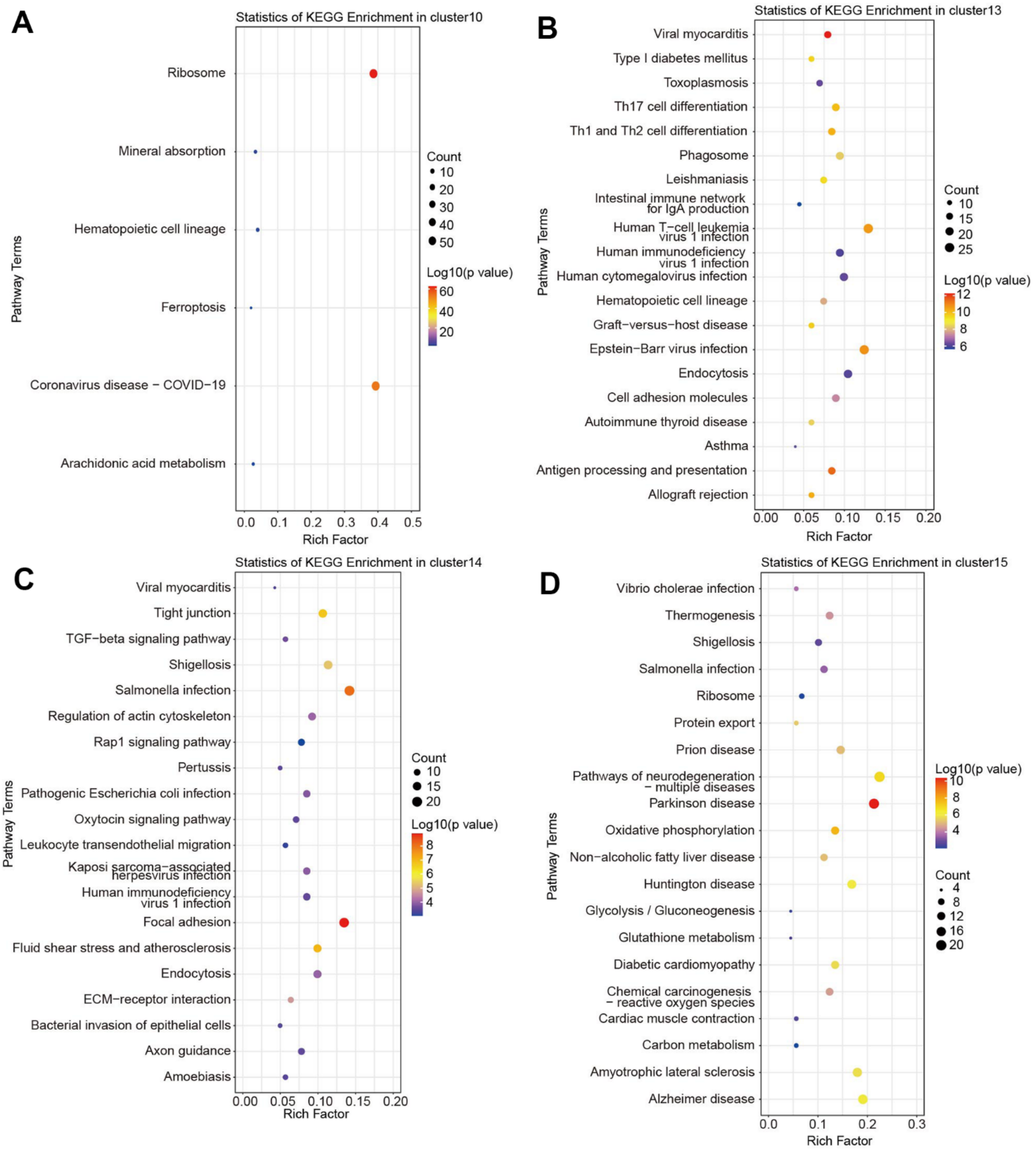

Supplementary Figure 2. The results of KEGG enriched analysis in cluster10 (**A**), cluster13 (**B**), cluster14 (**C**) and cluster15 (**D**).

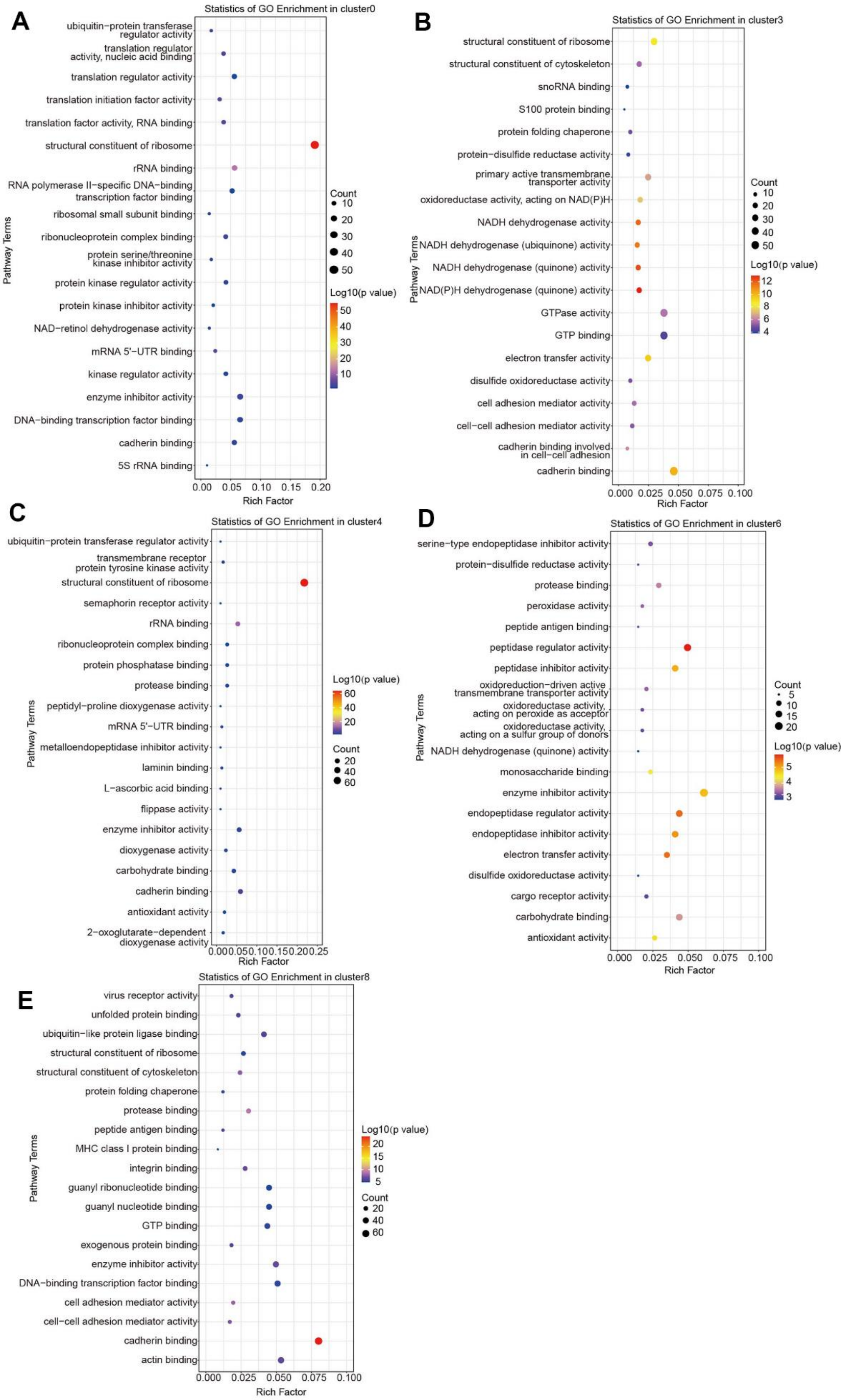

Supplementary Figure 3. The results of GO enriched analysis in cluster0 (**A**), cluster3 (**B**), cluster4 (**C**), cluster6 (**D**) and cluster8 (**E**).

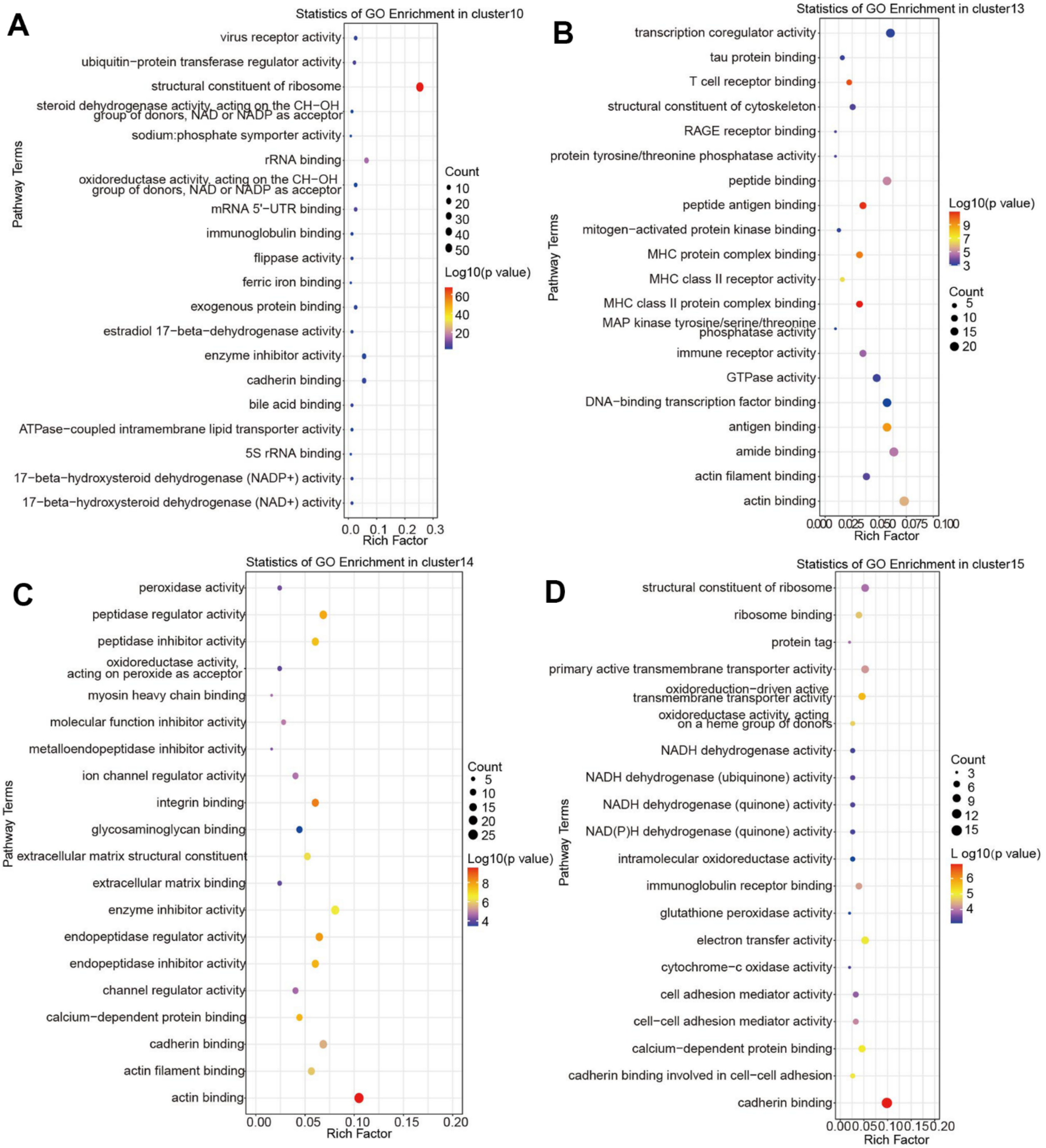

Supplementary Figure 4. The results of GO enriched analysis in cluster10 (**A**), cluster13 (**B**), cluster14 (**C**) and cluster15 (**D**).

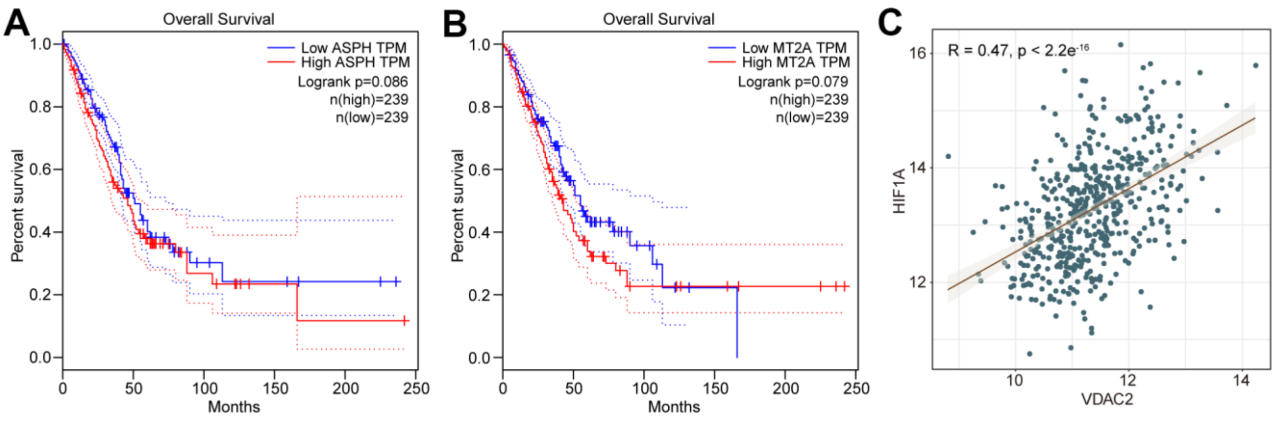

Supplementary Figure 5. The overall survival rate of patients with high or low *ASPH* (**A**) and *MT2A* (**B**) expression. (**C**) The correlation between *VDAC2* expression and hypoxia-inducible factor 1-α (HIF-1α).
