# Supplementary material for: Single-cell and bulk RNA sequencing data jointly reveals VDAC2’s impacts on prognosis and immune landscape of NSCLC
Source: Aging (Albany NY). 2024 Feb 20;16(4):3160–84. doi: 10.18632/aging.205517 (PMC10929798; doi:10.18632/aging.205517)
Supplement: Supplementary Tables [file aging-16-205517-s002.pdf]

# SUPPLEMENTARY TABLES

**Supplementary Table 1. Prognosis associated genes between C1 and C2 groups.**

| Gene | HR | z | P-value | Lower | Upper |
|---|---|---|---|---|---|
| LDHA | 1.360890939 | 3.707311499 | 0.000209471 | 1.156309886 | 1.601667659 |
| GAPDH | 1.311404491 | 3.57007769 | 0.000356875 | 1.130054847 | 1.521856875 |
| TPI1 | 1.230060274 | 2.335626069 | 0.019510739 | 1.033864827 | 1.463487527 |
| VDAC2 | 1.214727042 | 1.977002413 | 0.048041357 | 1.001677835 | 1.473090183 |
| ALDOA | 1.209959033 | 2.281359892 | 0.022527159 | 1.027213353 | 1.425215957 |
| CTSL | 1.178130335 | 2.811213704 | 0.004935499 | 1.050891073 | 1.320775409 |
| MIF | 1.174266645 | 2.177645446 | 0.029432443 | 1.01618788 | 1.356936234 |
| PLAUR | 1.145686306 | 2.284546906 | 0.022339409 | 1.019510999 | 1.287477147 |
| ERRFI1 | 1.144607836 | 2.30380039 | 0.021233851 | 1.020362213 | 1.283982375 |
| ASPH | 1.134771595 | 2.312315735 | 0.02076029 | 1.019452455 | 1.263135487 |
| MT2A | 1.11485957 | 2.044269965 | 0.040926885 | 1.004494045 | 1.23735115 |
| SCGB1A1 | 0.957232918 | -2.496704198 | 0.012535347 | 0.924945346 | 0.99064757 |
| SFTPC | 0.951924195 | -3.082844116 | 0.002050325 | 0.922568243 | 0.982214248 |
| AQP5 | 0.950860677 | -2.394798961 | 0.01662948 | 0.912446049 | 0.990892588 |
| SCGB3A2 | 0.948836286 | -2.632648622 | 0.008472195 | 0.912453137 | 0.986670177 |
| PIGR | 0.943784359 | -2.540433142 | 0.011071526 | 0.902582536 | 0.986866996 |
| SCGB3A1 | 0.933044378 | -3.340134876 | 0.000837377 | 0.895862206 | 0.971769772 |
| CCL19 | 0.93144917 | -2.037018444 | 0.041648194 | 0.869931265 | 0.997317365 |
| IGHA1 | 0.925143464 | -2.027833339 | 0.04257726 | 0.858122155 | 0.997399291 |
| FOLR1 | 0.923837419 | -2.665116957 | 0.007696155 | 0.871553474 | 0.979257845 |
| IGKC | 0.923072168 | -2.202464509 | 0.027632513 | 0.859605156 | 0.991225123 |
| SLC22A31 | 0.919273438 | -2.949283292 | 0.003185119 | 0.86926396 | 0.972160002 |
| C4BPA | 0.917551185 | -3.465879222 | 0.000528501 | 0.873972233 | 0.963303117 |
| SFTA1P | 0.914684476 | -2.882207165 | 0.003949 | 0.860864799 | 0.971868859 |
| C7 | 0.913936967 | -2.854753547 | 0.004307024 | 0.859177392 | 0.972186638 |
| SFTPB | 0.911886553 | -4.239856378 | 2.24E-05 | 0.873821241 | 0.951610061 |
| SFTPD | 0.909237414 | -3.544634867 | 0.000393157 | 0.862637628 | 0.958354526 |
| C16orf89 | 0.907997902 | -3.837446851 | 0.00012432 | 0.864324474 | 0.953878103 |
| NAPSA | 0.906894162 | -4.10130205 | 4.11E-05 | 0.865512626 | 0.950254215 |
| AQP3 | 0.906421516 | -2.681734804 | 0.00732415 | 0.843615789 | 0.973903021 |
| IGLC3 | 0.903923499 | -2.777570168 | 0.005476701 | 0.841737049 | 0.970704204 |
| RGS1 | 0.902500596 | -2.008603703 | 0.044579178 | 0.816533231 | 0.997518894 |
| DCN | 0.898596825 | -2.026246759 | 0.042739504 | 0.810305431 | 0.996508506 |
| HLA-DQB1 | 0.892443129 | -2.699060479 | 0.006953554 | 0.821663248 | 0.969320144 |
| GDF15 | 0.887924503 | -2.8459562 | 0.004427828 | 0.818132143 | 0.963670637 |
| IL7R | 0.887066616 | -2.420111186 | 0.015515762 | 0.805022091 | 0.977472781 |
| TRAC | 0.885218608 | -2.124717296 | 0.033610209 | 0.79105529 | 0.990590664 |
| TRBC2 | 0.885124367 | -2.171268029 | 0.029910918 | 0.792804386 | 0.988194767 |
| HLA-DRB5 | 0.884982441 | -3.061262949 | 0.002204054 | 0.818388905 | 0.956994793 |
| HLA-DPA1 | 0.884854804 | -2.594201323 | 0.009481095 | 0.806738717 | 0.970534832 |
| LTB | 0.883876998 | -2.522370393 | 0.01165669 | 0.803038818 | 0.972852782 |
| SPARCL1 | 0.882290731 | -2.183385667 | 0.029007421 | 0.788476734 | 0.987266841 |
| CXCL17 | 0.879437443 | -4.576053762 | 4.74E-06 | 0.832352798 | 0.929185579 |
| LST1 | 0.87873819 | -2.369291868 | 0.017822183 | 0.789619834 | 0.977914653 |
| MS4A7 | 0.878056597 | -2.39953259 | 0.016416018 | 0.78957082 | 0.976458815 |
| CYB5A | 0.877396939 | -2.253662039 | 0.024217437 | 0.783059806 | 0.983099097 |
| A2M | 0.876367584 | -2.350669233 | 0.018739684 | 0.785052556 | 0.97830411 |
| MFAP4 | 0.875952513 | -3.015423202 | 0.002566209 | 0.803700233 | 0.95470024 |
| CD79A | 0.875236415 | -3.363419537 | 0.000769833 | 0.809841682 | 0.945911774 |
| HLA-DRB1 | 0.874978348 | -2.7879849 | 0.005303702 | 0.796564948 | 0.961110718 |
| SFTA3 | 0.874968709 | -4.957668635 | 7.13E-07 | 0.829965145 | 0.922412521 |

| | | | | | |
|---|---|---|---|---|---|
| SOD3 | 0.873822967 | -2.512205712 | 0.011997913 | 0.786544699 | 0.970785994 |
| ACP5 | 0.873398978 | -2.059267081 | 0.03946866 | 0.767821436 | 0.993493719 |
| TSTD1 | 0.872882353 | -1.979854526 | 0.047719877 | 0.762964996 | 0.99863507 |
| HLA-DQA1 | 0.872548687 | -3.01019349 | 0.002610813 | 0.798430819 | 0.953546874 |
| CD74 | 0.871151342 | -2.760652055 | 0.00576861 | 0.789882007 | 0.960782312 |
| HLA-DRA | 0.870665571 | -2.854035064 | 0.004316777 | 0.791671919 | 0.957541272 |
| CD3D | 0.869754364 | -2.535314672 | 0.011234636 | 0.780811569 | 0.968828696 |
| CD48 | 0.869151053 | -2.609124888 | 0.009077411 | 0.782246932 | 0.965709831 |
| TSC22D3 | 0.868276687 | -2.231458122 | 0.025650799 | 0.766972058 | 0.982962022 |
| WFDC2 | 0.865746406 | -3.525547716 | 0.000422608 | 0.799068842 | 0.937987817 |
| HLA-DPB1 | 0.865639757 | -2.931664764 | 0.003371505 | 0.786038805 | 0.953301777 |
| IFT57 | 0.864129833 | -2.397057713 | 0.01652732 | 0.766871432 | 0.973723022 |
| CD37 | 0.864018055 | -2.565435951 | 0.010304624 | 0.772728572 | 0.9660924 |
| RNASE1 | 0.86325175 | -3.302698273 | 0.000957594 | 0.791113288 | 0.941968231 |
| BCAM | 0.862131242 | -2.664016675 | 0.007721374 | 0.772989487 | 0.961552895 |
| PTGDS | 0.861585425 | -3.315587512 | 0.000914507 | 0.788952486 | 0.940905135 |
| CD69 | 0.861299386 | -2.906354643 | 0.003656667 | 0.778796352 | 0.952542511 |
| HNMT | 0.856921261 | -2.304196835 | 0.021211597 | 0.751449934 | 0.977196235 |
| HLA-DMB | 0.854469141 | -3.03274766 | 0.002423382 | 0.771887532 | 0.945885874 |
| WSB1 | 0.852954777 | -2.600305038 | 0.009314093 | 0.756592281 | 0.961590371 |
| SNHG7 | 0.849665238 | -2.273041308 | 0.023023691 | 0.738313395 | 0.977811078 |
| NUPR1 | 0.848438019 | -2.911106298 | 0.003601515 | 0.759560035 | 0.947715834 |
| ID2 | 0.84258859 | -2.347495228 | 0.018900115 | 0.730315876 | 0.972121181 |
| CXCR4 | 0.842309652 | -2.607587163 | 0.009118285 | 0.740377932 | 0.958274849 |
| MGP | 0.841554756 | -3.26310677 | 0.00110198 | 0.758723601 | 0.933428729 |
| LTA4H | 0.840710894 | -2.086643419 | 0.036920379 | 0.714279236 | 0.989521704 |
| TRBC1 | 0.837212032 | -3.285221497 | 0.001019022 | 0.753007112 | 0.930833157 |
| TMSB4X | 0.833258015 | -2.299083481 | 0.021500199 | 0.713253948 | 0.973452612 |
| CD47 | 0.830496348 | -2.424255386 | 0.015339814 | 0.714700073 | 0.965054028 |
| EPHX1 | 0.828418415 | -3.51881723 | 0.000433475 | 0.745959504 | 0.919992394 |
| ALDH2 | 0.826400318 | -2.974475226 | 0.002934902 | 0.728827901 | 0.93703532 |
| ASAH1 | 0.823374798 | -2.436539778 | 0.014828538 | 0.704212712 | 0.962700681 |
| HLA-DMA | 0.823143292 | -3.491285992 | 0.000480701 | 0.737945727 | 0.918177117 |
| ATP6V0D1 | 0.822638322 | -2.070159278 | 0.038437431 | 0.683803519 | 0.989661197 |
| CTSZ | 0.822143977 | -2.46294847 | 0.013779972 | 0.703501686 | 0.960794739 |
| SSR4 | 0.820126991 | -2.39091454 | 0.016806464 | 0.697083361 | 0.964889308 |
| NPC2 | 0.818435795 | -3.507431274 | 0.000452455 | 0.731745627 | 0.915396178 |
| VAMP8 | 0.816019376 | -2.364851588 | 0.018037298 | 0.689475368 | 0.965788849 |
| CTSH | 0.815403585 | -3.791665484 | 0.00014964 | 0.733769919 | 0.906119191 |
| COX14 | 0.814171368 | -2.16454563 | 0.030422499 | 0.675881142 | 0.980756787 |
| C14orf28 | 0.811471679 | -1.99542611 | 0.045996426 | 0.660935533 | 0.996294271 |
| KLRB1 | 0.811261893 | -3.496017517 | 0.000472258 | 0.721496564 | 0.912195417 |
| FBP1 | 0.809136416 | -3.522662073 | 0.000427236 | 0.719194285 | 0.910326671 |
| SMDT1 | 0.80182364 | -2.328189804 | 0.019902027 | 0.66577667 | 0.96567089 |
| FCGRT | 0.783363551 | -3.180936097 | 0.001468 | 0.673950311 | 0.91053961 |
| HMGN3 | 0.782802046 | -3.044670394 | 0.002329354 | 0.668639276 | 0.916456848 |
| CIRBP | 0.7817752 | -2.967341056 | 0.003003875 | 0.664448321 | 0.919819411 |
| ISCU | 0.755610674 | -2.501133314 | 0.012379657 | 0.606636824 | 0.941168535 |
| AKR1A1 | 0.749640083 | -2.631956588 | 0.008489471 | 0.604864718 | 0.929067671 |
| PEBP1 | 0.745189336 | -3.066893752 | 0.002162957 | 0.61749848 | 0.899285043 |

**Supplementary Table 2. The clinical features of NSCLC patients.**

| Sex | Age | Tumor size (cm×cm×cm) | T | N | M | Differentiation grade |
|---|---|---|---|---|---|---|
| Male | 45 | 2.5*2*1.5 | 4 | 2 | 1a | medium-low differentiated adenocarcinoma |
| Female | 56 | 3.5*3*3 | 2a | 2 | 0 | medium-low differentiated adenocarcinoma |
| Male | 62 | 5*4.5*4 | 3 | 1 | 0 | medium-differentiated adenocarcinoma |
| Female | 66 | 3.1*2.6*2.2 | 3 | 0 | 0 | medium-differentiated adenocarcinoma |
| Female | 57 | 4.5*3.5*3 | 2b | 2 | 0 | medium-differentiated adenocarcinoma |
| Male | 56 | 6.5*4*4 | 3 | 0 | 0 | poorly differentiated adenocarcinoma |
| Male | 50 | 1*1*0.3+1.5*1*0.5 | 3 | 1 | 1a | poorly differentiated adenocarcinoma |
| Female | 48 | 1.1*0.9*0.3 | 1b | 0 | 0 | highly differentiated adenocarcinoma |
| Female | 68 | 1*1*0.7 | 2a | 0 | 0 | medium-differentiated adenocarcinoma |
| Female | 59 | 4.2*4.5*2.6 | 2b | 0 | 0 | medium-differentiated adenocarcinoma |
| Female | 55 | 2*1.5*1 | 2a | 0 | 0 | medium-differentiated adenocarcinoma |
| Male | 59 | 3.7*3.3*1.7 | 2a | 2 | 0 | medium-low differentiated adenocarcinoma |
| Male | 68 | 5*3.5*2 | 2b | 0 | 0 | medium-low differentiated adenocarcinoma |
| Female | 60 | 2.6*1.8*1.5 | 2a | 0 | 0 | medium-differentiated adenocarcinoma |
| Male | 58 | 1.3*0.6*0.6 | 1b | 0 | 0 | highly differentiated adenocarcinoma |
